# Peer support interventions for young adults with inflammatory bowel diseases

**DOI:** 10.1016/j.hctj.2023.100018

**Published:** 2023-09-12

**Authors:** Sneha Dave, Amy Bugwadia, Sara Ahola Kohut, Sydney Reed, Mara Shapiro, Hilary K. Michel

**Affiliations:** a Generation Patient, USA; b Stanford University School of Medicine, USA; c Department of Gastroenterology, Hepatology and Nutrition, Hospital for Sick Children, Toronto, Canada; d Department of Psychiatry, University of Toronto, Canada; e Child Health Evaluative Sciences, SickKids Research Institute, Canada; f Division of Pediatric Gastroenterology, Hepatology and Nutrition, Nationwide Children's Hospital, Columbus, OH, USA; g Department of Pediatrics, The Ohio State Wexner Medical Center, Columbus, OH, USA

**Keywords:** Peer support, Young adults with inflammatory bowel disease, Adolescents with inflammatory bowel disease, Adolescents and young adults

## Abstract

The “Roundtable on Young Adults with Inflammatory Bowel Diseases (IBD)” is a year-long learning community organized by the Crohn’s and Colitis Young Adults Network with monthly discussions between healthcare professionals, patients, and community-based partners. The group aims to improve outcomes for the young adult IBD patient population. The roundtable discussion highlighted in this article centered on the critical importance of peer support for adolescents and young adults (AYA) with IBD. The discussion underscored the need for increased research in peer support, the need to further consider peer support as an intervention, and the value of multi-stakeholder, peer-led collaboration in reducing isolation for young adults with IBD. Additionally, the group identified areas of opportunity, such as developing more appropriate metrics and scales to prove the inherent value of peer support for young adults with IBD.

## 1 Introduction

The roundtable session on peer support for adolescents and young adults (AYA) with inflammatory bowel diseases (IBD) was designed to include a short presentation by two key opinion leaders in peer support, Dr. Sara Ahola Kohut, a psychologist with the IBD program at the Hospital for Sick Children in Toronto, Canada, and Mara Shapiro, a young adult patient with IBD. Dr. Ahola Kohut shared the clinical definition of peer support and major categories of peer support within the clinical context, including informational support, appraisal support, and emotional support. 1, 2 Dr. Ahola Kohut called attention to the iPeer-to-Peer Program model ^3^ and the unique capabilities of peer interactions to support the learning and practicing of coping and IBD self-management strategies in a developmentally sensitive and flexible manner. Ms. Shapiro underscored the value of peer support in her own journey navigating a diagnosis and shared that “By ‘prescribing’ peer support, you are allowing your patient to build skills that will help them help themselves.”

The discussion that followed included patients, carepartners (e.g., parents, guardians), researchers, and physicians and was intended to spur multi-stakeholder collaborations to increase research in peer support for young adults with IBD. The discussion questions included:

• What research or collaborative projects do you think are important to pursue in the peer intervention space?
• What barriers do you see keeping you or your practice from including peer-support interventions in your services?

## 2 Peer-support interventions are not a “one-size-fits-all” for young adult patients

Young adult patients undergo significant life changes, such as transitioning from pediatric to adult care, independent living, and entering higher education and/or the workplace. Isolation during this time can be pronounced, making the significance of peer relationships even more critical. Furthermore, adolescence and young adulthood are important periods for developing one’s sense of self, which traditionally occurs in the context of peer relationships.

Peer-support interventions provide young adult IBD patients with the opportunity to meet others and to build self-efficacy in relation to disease knowledge, medical management, navigating daily life with IBD, and planning for the future. 4, 5 Previous research has shown that increasing self-efficacy can also increase adolescents’ readiness to transition from pediatric to adult care, which can have positive psychosocial and clinical impacts on individuals with IBD. ^6^ Given this, the importance of identifying the right peer-support intervention cannot be overstated. Peer support can encompass a variety of different formats, as noted by Ms. Shapiro, including traditional support groups, open-ended discussions and virtual meetings, one-on-one peer mentoring, camps, online social communities, and disability-affinity groups for university students ^7^. Each of these forms of peer support can serve different purposes, such as educational or emotional, and thus, no universal "one size fits all" support can be prescribed for all patients. Just as disease activity can wax and wane, individuals living with IBD can also have fluctuating psychosocial support needs. Roundtable participants thus emphasized the importance of making various peer-support options known and available to AYAs with IBD in order to empower patients to engage in what feels right for them at the current moment and to recognize that the “right” intervention for that individual can change over time. Given the paucity of literature in this area, future peer-support research should identify best practices to guide clinicians in identifying which peer-support interventions should be offered to which patient at the appropriate time.

The potency of peer support for care partners (i.e. parents, guardians, siblings, etc.) was also recognized, acknowledging their concurrent journey as young adults transition toward self-managing their care. As patients transition to adult care, the care partner's influence on their perception of and identity with the condition, whether positive or negative, may become more apparent. As noted by one medical professional during the roundtable discussion, peer support for care partners may decrease “miscarried helping” in caregivers who might be engaging in problematic behaviors, and, for young adult patients, exposure to other patients of a similar age can be empowering and enable growth during this integral period.

## 3 “Peer support can heal the soul, even if it is not proven to heal the gut”: Research in peer-support needs to be creative and look at outcomes differently

The unique psychosocial challenges experienced by youth with IBD have been well-documented in the literature and include an increased risk of anxiety, depression, body image dissatisfaction, and social and disease-related stigma. 8, 9 These, in turn, have been associated with challenges with self-efficacy, IBD symptoms, and risk of disease recurrence. 10, 11, 12 There is evidence that peer-support programs, which involve patients connecting with others possessing similar disease experiences, can improve mental health outcomes and increase patients' self-efficacy, enabling patients to better self-manage their condition. 13, 14, 15 However, there is limited research assessing peer-support interventions’ impact, and specifically, the correlation between potential psychosocial and physiological benefits. Thus, it is critical to not only increase the number of longitudinal research studies in this area but to also ensure that the data collected are meaningful to clinicians and patients alike.

While mucosal healing and clinical markers of inflammation should be monitored, there is still significant value in interventions that improve outcomes such as quality of life, resilience, self-management, and self-confidence. However, the consensus among roundtable participants is that existing validated measures of psychosocial health and quality of life, while helpful, may not adequately capture the full extent of the impact of peer-support interventions. There is an inherent value in not feeling alone and the power of social connection, especially in AYA populations. Therefore, it is critical to assess non-traditional indicators to understand the multi-faceted impacts of peer-support interventions.

One identified solution included incorporating qualitative assessments, such as interviews with participants in peer-support programs, and adding open-ended questions to quality-of-life surveys. ^5^ These methods may be able to better capture what matters most to patients to ensure that the outcomes of these studies align with the lived experiences and goals-of-care for individuals with IBD. Similarly, intentionally incorporating patients into studies and clinic-based programs through the formation of Patient Advisory Boards (PAB) can achieve these goals.

## 4 Patients can and should co-lead peer-support intervention research

Community-based participatory research (CBPR) is a research framework that intentionally involves community members, in this case, IBD patients, in all aspects of the research design, implementation, and analysis processes. ^16^ CBPR has been identified as a best practice for advancing patient inclusion and driving equity in the IBD space and beyond. 17, 18 Not only does adopting a CBPR approach ensure that research questions are authentic to the community, but it has also been shown to increase the sustainability of the interventions being developed. Furthermore, patient input early on in research can enable outcomes to match the true needs of the community. ^19^

As an example of how CBPR can be used in practice, multiple roundtable participants mentioned the creation of a PAB as a clear opportunity for more patient-driven research. ^20^ In line with CBPR principles of treating patients as partners rather than solely research subjects, roundtable participants also emphasized that providing appropriate compensation for PAB members is key to advancing equity and increasing accessibility. This compensation can include providing meals during PAB meetings and gift cards to PAB members. Moreover, recognizing that diverse, intersectional perspectives are crucial but often lacking in these spaces, care must be taken to intentionally include patients from minoritized communities and to make PAB opportunities accessible. This can include reimbursement of travel expenses (e.g., for gas or public transportation), providing virtual participation options, and scheduling meetings outside of traditional work/school hours. These recommendations for compensating PAB members are further echoed within published best practice guidelines on building and sustaining community-based programs and research. 21, 22

## 5 Peer-support research should include young adults from marginalized and underrecognized backgrounds

Young adults with IBD who participate in peer-support spaces may not be representative of the majority of young adult patients affected by the condition but rather, may hail from backgrounds for which there is easier access and greater acceptability. Recognizing this potential lack of representation and inclusion, roundtable participants shared concerns that current peer support research may not be representative of the unique needs of patients from marginalized and underrepresented backgrounds. This includes young adult patients with lower socioeconomic status, those experiencing housing insecurity or homelessness, incarcerated individuals, youth currently in or transitioning out of the foster care system, those living in rural areas, as well as Black, Indigenous, and people of color.

In addition to increasing intentionality and accessibility for these patients to be involved in PABs, care must be taken to highlight the work that patients are already doing within their own communities. For example, one roundtable participant drew attention to the efforts of the IBD Patient Support Foundation (India), an organization founded and led by people in India living with IBD, in creating a message-based, peer-support network for women. This is particularly important from a global perspective, as peer support has multiple cultural considerations, such as societal hesitancies with the disclosure of having a chronic medical disability. 23, 24, 25

## 6 Conclusion

Peer-support interventions have the potential to improve a variety of outcomes for AYAs with chronic diseases, but currently selected outcomes may not be capturing the comprehensive value of these programs. Patients should be leading the research in peer-support interventions and be active members of peer-support research teams. In addition, young adults with IBD have needs that necessitate flexibility in peer-support approaches. Future work should consider diverse peer-support models and allow young adults with IBD to choose their preferred format.

## Declaration of Competing Interest

The authors declare no competing interests.

## Data Availability

No data was used for the research described in the article.
